# Electro-Osmotic Behavior of Polymeric Cation-Exchange Membranes in Ethanol-Water Solutions

**DOI:** 10.3390/e22060692

**Published:** 2020-06-20

**Authors:** V. María Barragán, Juan P. G. Villaluenga, Víctor Morales-Villarejo, M. Amparo Izquierdo-Gil

**Affiliations:** Department of Structure of Matter, Thermal Physics and Electronics, Complutense University of Madrid, 28040 Madrid, Spain; jpgarcia@ucm.es (J.P.G.V.); victorv11@hotmail.com (V.M.-V.); amparo@ucm.es (M.A.I.-G.)

**Keywords:** cation-exchange membrane, electro-osmotic permeability, streaming potential, ethanol, Onsager reciprocity relations

## Abstract

The aim of this work is to apply linear non-equilibrium thermodynamics to study the electrokinetic properties of three cation-exchange membranes of different structures in ethanol-water electrolyte solutions. To this end, liquid uptake and electro-osmotic permeability were estimated with potassium chloride ethanol-water solutions with different ethanol proportions as solvent. Current–voltage curves were also measured for each membrane system to estimate the energy dissipation due to the Joule effect. Considering the Onsager reciprocity relations, the streaming potential coefficient was discussed in terms of ethanol content of the solutions and the membrane structure. The results showed that more porous heterogeneous membrane presented lower values of liquid uptake and streaming potential coefficient with increasing ethanol content. Denser homogeneous membrane showed higher values for both, solvent uptake and streaming coefficient for intermediate content of ethanol.

## 1. Introduction

Membrane processes are widely studied for numerous applications of practical interest. Linear non-equilibrium thermodynamics is one of the theoretical approaches used to describe transport phenomena in membrane systems [1]. Within this approach, each thermodynamic flow, *J_i_*, depends on every thermodynamic force, *X_k_*, acting in the system,

(1) $$J_{i}=\underset{k}{\sum }L_{ik}X_{k}$$

where *L_ik_* are the so-called phenomenological coefficients. The Onsager relation holds *L_ik_* = *L_ki_*. In studying a system, only one thermodynamic force acts usually over it, or one of the thermodynamic flows becomes null in order to determine the different phenomenological coefficients. From these known coefficients and the linear phenomenological Equation (1), it is possible to determine the total flow through the system when various forces act over it.

When a charged membrane separating two aqueous-organic electrolyte solutions is subject to one or more driving forces, transport electrokinetic phenomena can originate through the membrane, indicating that mass and electric charge transport processes can be coupled. For instance, a pressure difference across a membrane is able to drive both a volume flux and an electric charge flux through the membrane. When a charge membrane separates two electrolyte solutions, and a pressure difference is established between the two sides of the membrane, the solvent is forced to move across it along the pressure difference. A net charge is carried along, as a consequence of the excess of counter-ions with respect to the co-ions, towards the low-pressure membrane side. The low-pressure side acquires the same charge as the counter-ions, while the high-pressure membrane side acquires the charge of the fixed groups. As a consequence, an electric potential difference, named streaming potential, appears between both sides of the membrane. When an electric current circulates through a charged membrane, ion transfer is accompanied by an associated solvent flow. This phenomenon is known as electro-osmosis. Electro-osmotic transport involves solvent transported either by an association with the transported ion, such as hydration sphere, or by hydrodynamic pumping due to the movement of ions and associated solvent molecules.

Electrokinetic effects-based techniques are often used to measure physical and chemical properties of porous and charged layers, because they reveal information on the physicochemical state of an interface in contact with an electrolytic solution. Thus, streaming potential measurements has been used to investigate the surface characteristics of ultrafiltration membranes, and to determine the interactions between the foulant and the membrane [2,3].

The coupling between solvent and charge flows, reflected by the Onsager reciprocity principle, also points to the use of electrokinetic effects for energy conversion. Electrokinetic effects in membrane systems can be utilized for conversion between mechanical and electrical energies [4,5,6,7,8,9,10]. These systems could also be used as electric generators or electrokinetic pumps [11,12,13], and the efficiency of the process strongly depends on the membranes’ properties. Due to their potential applications, they have increasingly attracted more and more attention. Obtaining efficient, low-cost electrokinetic generators and pumps for small microscale applications is a challenge. The electrokinetic behavior in organic media is important for using these devices in biomedical applications [12], and also in the study of direct ethanol fuel cells, where a pressure difference can exist between both the anode and cathode [14], affecting fuel cell performance.

In this study, linear non-equilibrium thermodynamics is applied to study electrokinetic properties of cation-exchange membranes. With this aim, the liquid uptake and the electro-osmotic permeability were experimentally determined for several commercial cation-exchange membranes of different structures and electro-chemical properties in ethanol-water media.

## 2. Model Equations

Electrokinetic phenomena originate as a consequence of the interaction between electrical charge and matter fluxes through an ion-exchange membrane. Due to the coupling between processes, the non-equilibrium thermodynamics was satisfactorily used in a study by Rastogi and colleagues [15]. Coupling means that a volume flux through a membrane may arise from an electric potential difference across the membrane, and an electric current can originate through a membrane by a pressure difference across the membrane. The total entropy production can be written as

(2) $$℘=J_{V}\left(\frac{\Delta P}{T}\right)+I\left(\frac{\Delta \varphi }{T}\right)$$

In Equation (2), Δ*P* and Δ*φ* are the pressure and electric potential differences, respectively, *J_V_* is the volume flow, and *I* is the electric current. In this approach, the transport equations for matter and electric charge through a membrane are

(3) $$J_{V}=L_{11}\Delta P+L_{12}\Delta \varphi$$

(4) $$I=L_{21}\Delta P+L_{22}\Delta \varphi$$

where the temperature is included in the phenomenological coefficients. The coefficients *L*_11_ and *L*_22_ are, respectively, the hydraulic permeability and the electric conductance,

(5) $${\left(\frac{J_{V}}{\Delta P}\right)}_{\Delta \varphi =0}=L_{11}$$

(6) $${\left(\frac{J_{V}}{\Delta P}\right)}_{\Delta \varphi =0}=L_{11}$$

whereas *L*_12_, which must be equal to *L*_21_ according to the Onsager reciprocity relation, is the electrokinetic coefficient. In the absence of pressure difference and taking into account Equations (3) and (4), the following relations can be obtained:

(7) $${\left(\frac{J_{V}}{\Delta \varphi }\right)}_{\Delta P=0}=L_{12}$$

(8) $${\left(\frac{J_{V}}{I}\right)}_{\Delta P=0}=\frac{L_{12}}{L_{22}}=W$$

Equations (7) and (8) give expressions for the electrokinetic coefficient, *L*_12_, and the so-called electro-osmotic permeability, *W*, in terms of the phenomenological coefficients. Accordingly, both *L*_12_ and *W* can be determined from measurements of the electro-osmotic flow. On the other hand, coupling can also be studied in terms of the so-called pressure or streaming potential coefficient, *β*, which can be defined from Equations (3) and (4), as follows:

(9) $${\left(\frac{\Delta \varphi }{\Delta P}\right)}_{I=0}=-\frac{L_{21}}{L_{22}}=\beta$$

This parameter quantifies the conversion of mechanics energy, from an applied pressure difference, in electric energy using the membrane system. According to Onsager’s reciprocity relations, which imply that *L*_12_ = *L*_21_, Saxén’s law establishes the equality between the electro-osmotic permeability and the pressure coefficient,

(10) β=−W

On the other hand, in the absence of a pressure difference between the two sides of the membrane, the energy dissipated per unit time, *Φ*, in the system is given by

(11) $$\Phi =T℘=I\Delta \varphi =I^{2}R_{cell}$$

where $R_{cell}$ is the electrical resistance in the membrane cell.

## 3. Experimental

### 3.1. Membranes and Materials

To carry out this study, three different commercial cation-exchange membranes were used. Their main characteristics are shown in Table 1. Nafion 117 (hereafter named NF117), produced by Dupont Inc. (Wilmington, DE, USA), is a homogeneous membrane that consists of a polytetrafluorethylene backbone and long fluorovinyl ether pendant side chains regularly spaced and ending in sulfonated ionic groups. There are no cross-links between the polymers. MK-40 membrane (hereafter named MK40), manufactured by Shchekinoazot (Russia), is a heterogeneous membrane that consists of composite material formed from the cation-resin KU-2, a polystyrene matrix cross-linked with divinylbenzene and fixed groups, polyethylene and nylon. CR67 HMR 412 (hereafter named CR67), manufactured by Ionics Inc. (Massachusetts, MA, USA) is a heterogeneous crosslinked sulfonated copolymer of vinyl compounds cast in homogeneous films on synthetic reinforced fabrics.

**Figure 1:**
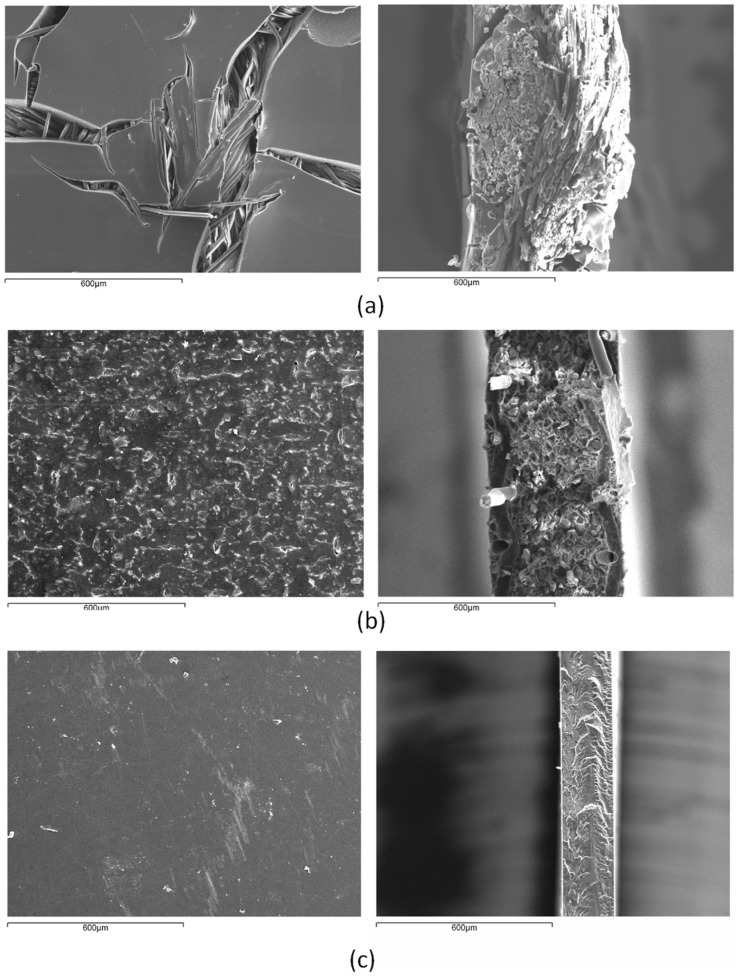
Scanning electron microscopy (SEM) images of the surface (left) and cross section (right) of the membranes used in this work (Spanish National Centre for Electron Microscopy, ICTS). (**a**) CR67; (**b**) MK40; (**c**) NF117.

The selected membranes possess similar electric properties but different morphology for the purpose of analyzing the influence of the membrane structure on the properties studied. NF117 was selected as the reference membrane for direct alcohol fuel cells, and MK40 and CR67 were references for electrodialysis. Figure 1 shows scanning electron microscopy (SEM) images of the surface and cross section of the unused dry membrane samples analyzed in this study. The images show important morphological differences between the different membrane samples. Heterogeneous membranes can be considered porous membranes, whereas the homogeneous NF117 membrane is considered a dense membrane [16,17]. This perfluorinated membrane is generally assumed to be macroscopically homogeneous, although it has been shown to exhibit certain inhomogeneity in the structure and present microheterogeneity. Heterogeneous MK40 membrane is prepared by the inclusion of a finely ground ion-exchange resin in the polyethylene (PE) binder. The greater part of the membrane surface in the dry state is screened with the non-conducting inert material (PE), and the active ion conducting phase where ionogenic groups are localized (ion exchanger phase) occupies a small area. Heterogeneous CR67 membrane presents a “sandwich” structure with the crosslinked sulfonated copolymers between the two homogeneous films.

In the experiments, 0.01 M potassium chloride solutions with a mixture of water and ethanol at different ethanol percentages (25 wt. %, 50 wt. %, 75 wt.% and 99 wt.%) were used as solvent. Pure pro-analysis grade chemicals and distilled pure water were used. In order to prevent bubble formation during the measurement process, the alcohol-water solutions were degassed before carrying out the measurements.

### 3.2. Measurement of the Electro-Osmotic Flow and Current-Voltage Curves

The experimental device used in this study and the methodology employed were similar to those described in earlier publications [18]. The experimental set-up basically consisted of two glass chambers separated by the membrane under study, which was vertically positioned between the two chambers. Experiments were performed with KCl-containing aqueous ethanol solutions. The concentration of KCl was kept fixed at 0.01 M. In contrast, the proportion of ethanol was varied as 25 wt.%, 50 wt.%, and 75 wt.% in the ternary solutions. The membranes had the same equilibrating solution on both sides. The membrane effective area exposed to the flow was 5.7 × 10^−4^ m^2^.

The cell is provided with a chain-driven cell magnetic stirrer assembly, which permits stirring of both solutions. To measure electro-osmotic fluxes, an Ag/AgCl electrode with a large active surface was introduced in each chamber on both sides of the membrane to inject an electric current. The experimental device was modified for using the four-electrodes configuration in the measure of the corresponding current–voltage curve. To this end, other Ag/AgCl electrodes were introduced in each chamber to measure the direct current (DC) voltage, together with the electrodes used to inject the current. In this case, the electrodes consisted of two linear Ag wires of approximately 4 mm in length and 1 mm in diameter and they were also prepared by the usual method [19].

One shaped capillary tube was introduced in each chamber, in such a way that the horizontal portions of the tubes were at the same height, to prevent pressure differences between the two chambers. All the experiments were carried out under isothermal conditions at 298 K by immersing the cell in a water thermostatted bath. The temperature was constant within ±0.1 K.

The volume flow was determined by measuring the time displacement of the solution meniscus in the capillary tubes connected to each chamber when a constant electric current, *I*, was circulated through the membrane system.

To determine the volume change rate, the volume change due to the electrochemical reactions at the Ag/AgCl electrodes must be taken into account. In this case, the volume change of the cathodic and anodic compartments, Δ*V_c_*, and Δ*V_a_*, respectively, can be expressed as:

(12) $$\Delta V_{c}=J_{V}+\frac{I}{F}\left(V_{Ag}-V_{AgCl}\right)$$

(13) $$\Delta V_{a}=-J_{V}+\frac{I}{F}\left(V_{AgCl}-V_{Ag}\right)$$

where *F* is the Faraday constant and *V*_Ag_ and *V*_AgCl_ are the partial molar volumes of Ag and AgCl, respectively. Taking into account that $V_{AgCl}-V_{Ag}=$ 15.5 cm^3^/mol, we have

(14) $$J_{V}\left(ml/s\right)=\frac{\left|\Delta V_{a}\right|+\left|\Delta V_{c}\right|}{2}+\left(1.6\times 10^{-4}\right)I$$

The corrective term taking into account the volume change due to the chemical reactions in the Ag/AgCl electrodes is indicated by the last term in the right-hand side of Equation (14).

In the measure of the current-voltage curves, a constant electric current was made to circulate through the membrane system, measuring the electric voltage when steady state conditions were reached.

It was checked that the electric current passing through the system was constant up to the hundredth or tenth part of mA during the measurement time. Once it reached the steady state, the electric voltage kept constant up to the hundredth part of V.

## 4. Results and Discussion

### 4.1. Liquid Uptake of the Membranes

**Figure 2:**
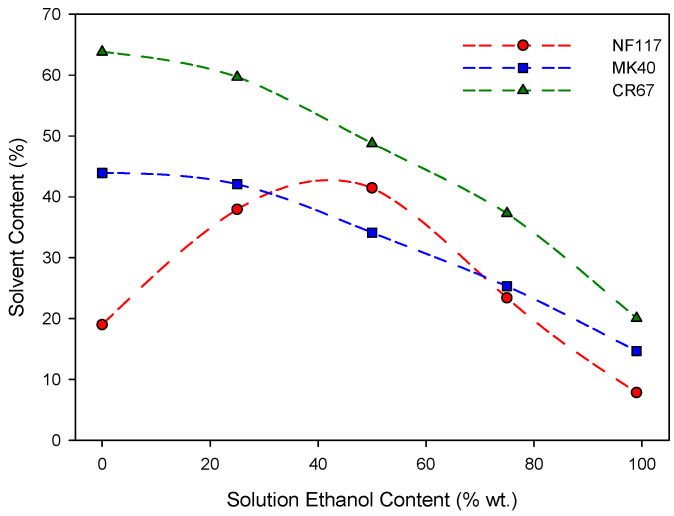
Liquid uptake of the membranes at different ethanol content in solution. Dotted lines are only a visual guide.

Membrane transport behavior is closely dependent on the amount of absorbed liquid. Therefore, it is important to know the solvent uptake properties of a membrane. For this reason, liquid uptake by the membranes was estimated by using the usual gravimetric method at 25.0 ± 0.1 °C. The values obtained are shown as a function of the ethanol content in solution in Figure 2. In general, higher liquid uptakes were observed for more porous heterogeneous membranes. In fact, a trend can be observed between uptake and dry membrane density, so that the higher the density the lower the liquid uptake. This trend only fails for homogeneous membranes at intermediate ethanol percentages. A different trend with the ethanol percentage was also observed between homogeneous NF117 and heterogeneous MK40 and CR67 membranes. Heterogeneous reinforced membranes showed a decrease in liquid uptake with the increase of the ethanol percentage on solvent. Homogeneous non-reinforced NF117 membrane showed a different trend, with an increase in the ethanol percentage for concentrations lower than approximately 50 wt.% and a decrease for higher ethanol concentrations. Previous studies have shown that the swelling properties of this membrane are strongly affected by the presence of alcohol in the solution [20]. A similar behavior was observed for these same membranes with 0.01 M LiCl and NaCl methanol-water ternary solutions [21].

From the liquid content of the membrane, *S*, the average number of liquid molecules per conducting functional group, *λ*, can be determined using the expression:

(15) $$\lambda =\frac{S}{100IECM_{s}}$$

where *M_s_* is the molar mass of the liquid sorbed. Values obtained with water and 99 wt.% ethanol are shown in Table 1. Higher values were observed with water, indicating a preferential water uptake, independently of the membrane structure. However, it is known that in binary water-alcohol solutions, in Nafion membranes, alcohol is preferentially taken by the membrane over water [22].

### 4.2. Determination of the Dissipated Energy

**Figure 3:**
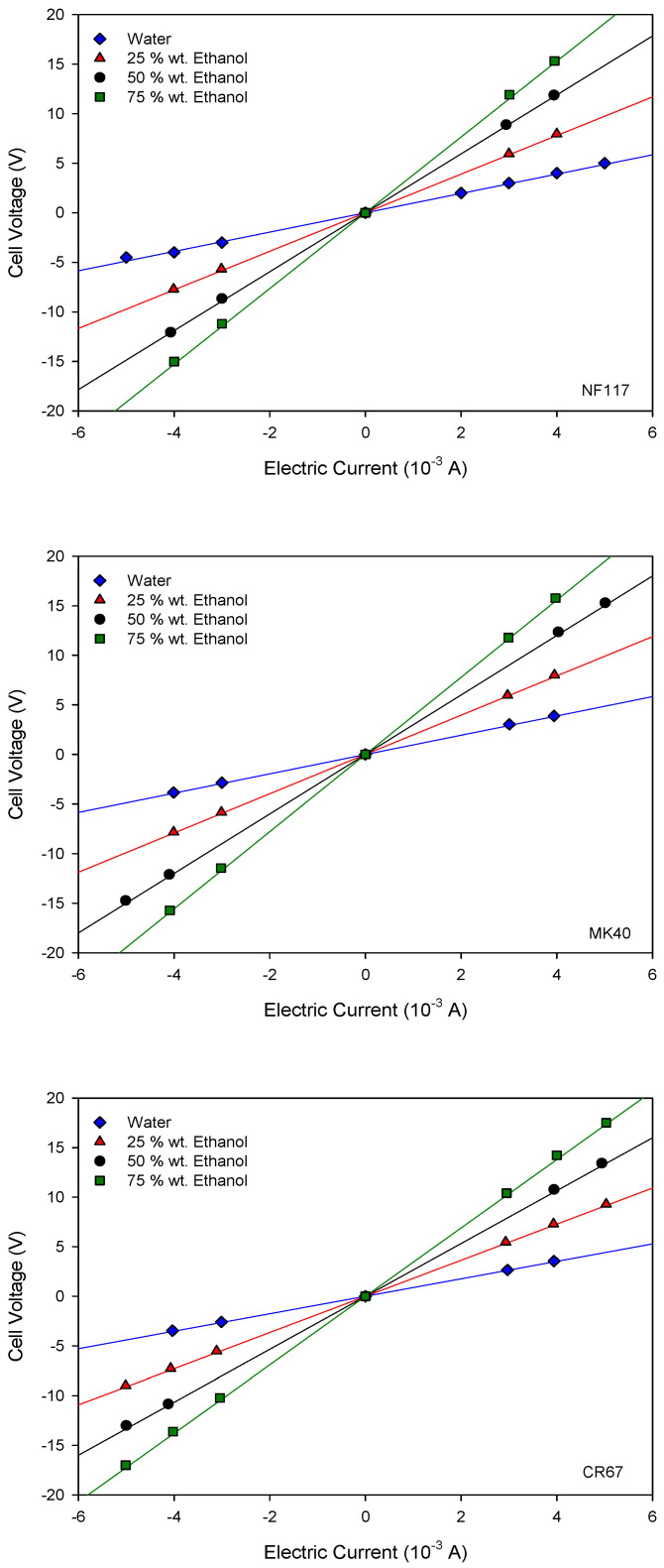
Current–voltage curves in 0.01 M KCl solution with different ethanol content in solvent. The name of the corresponding membrane is indicated at the bottom right of the figure.

Current–voltage curves were obtained for the different studied membranes. The results are shown in Figure 3.

**Figure 4:**
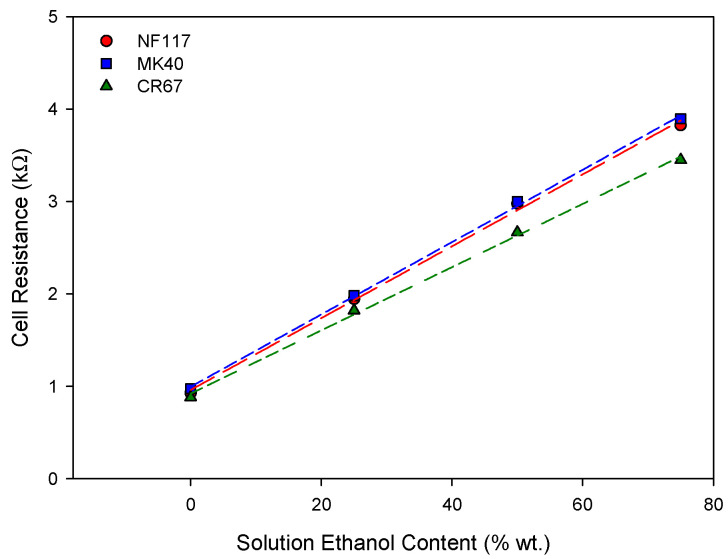
Cell resistance of the membrane systems in 0.01 M KCl solution with different ethanol percentages in solvent.

In agreement with Ohm’s law, a linear behavior was observed between the applied current and the cell voltage for all the measured systems. Accordingly, from the slope of these curves we can obtain the cell resistance for each membrane system with the different ethanol contents. The results are shown in Figure 4.

A linear behavior was also observed between the cell resistance and the percentage of ethanol in the solution for the three membranes. Table 2 shows the cell resistances in pure water and the slopes of the straight lines in Figure 4.

As can be observed, both the cell resistance in pure water and the rate of the increase with the ethanol percentage were similar for NF117 and MK40 membranes; the differences were practically within the experimental error. Slightly lower values were observed for CR67 membrane. The linear trend was probably due to the larger contribution of the total cell resistance and the solution contribution which was the same in the three studied membrane systems. Specific conductivity of ethanol-water potassium chloride solutions decreases with the increase of ethanol content, which would explain the increase in cell resistance. For 0.01 M KCl solutions used in this work, the molar conductivity decreased from 141 × 10^−4^ S m^2^ mol^−1^ with pure water as solvent to 52 × 10^−4^ S m^2^ mol^−1^ and 34 × 10^−4^ S m^2^ mol^−1^ with 40 wt.% and 79.5 wt.% ethanol-water mixtures as solvent, respectively [23]. This decrease is due to the decrease in the dielectric constant of the solvent with increasing ethanol content from the value for pure water of 78 to 72, 52, 41, and 22 for ethanol percentages in the binary water-ethanol mixtures of 25, 50, 75, and 100, respectively, and so, to the decrease in the ability of the solvent to dissolve the electrolyte [24]. The differences for CR67 membrane increase with increasing the percentage of ethanol, with indicates the influence of the membrane structure. The membrane resistance depends on the conductivity of the solutions in contact with it, so a similar dependence of the membrane resistance on the percentage of ethanol is expected. Thus, the results would indicate that the K^+^ form of the CR67 membrane possesses a higher electric conductivity and a lower influence of the ethanol percentage in the solvent.

**Figure 5:**
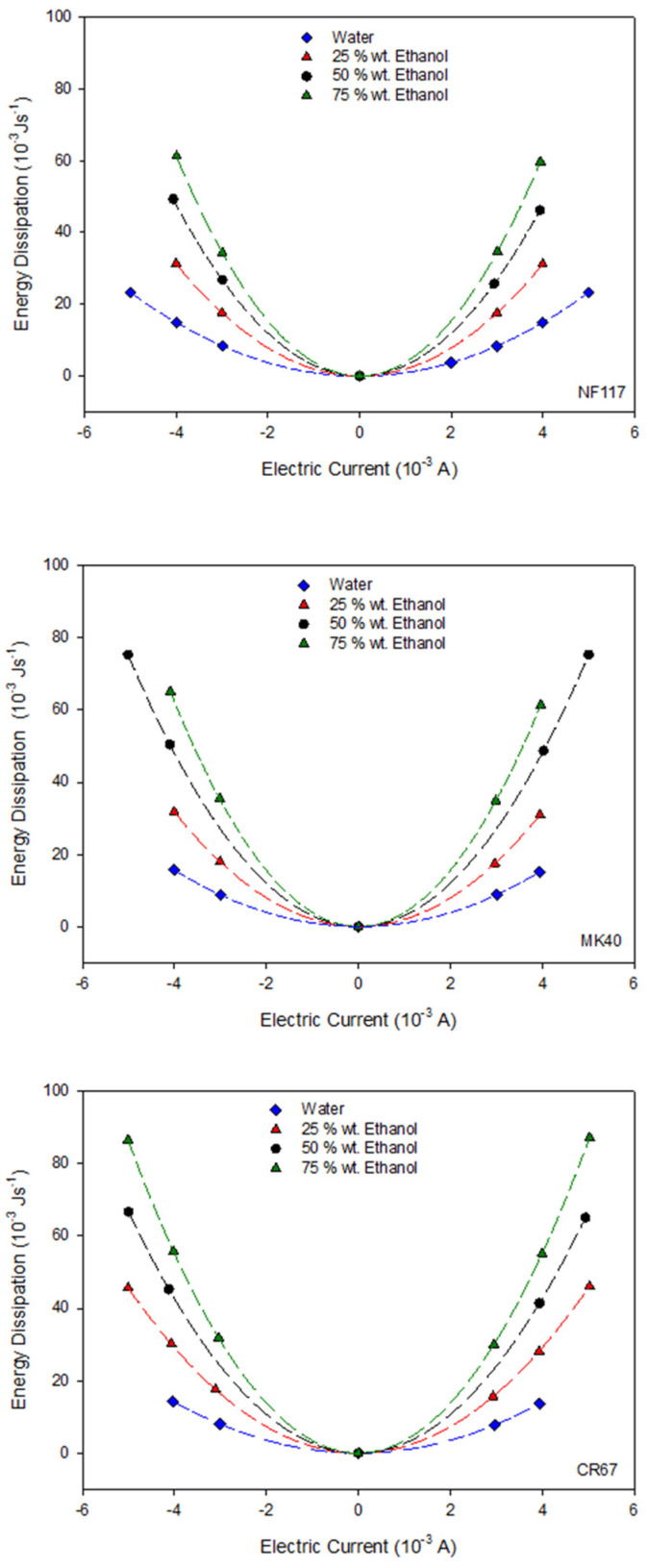
Energy dissipation at different electric current and different ethanol content in solvent for the three studied membranes. The name of the corresponding membrane is indicated at the bottom right of the figure.

From the cell resistance values, the dissipation energy due to the Joule effect can be estimated using Equation (11) for the three membrane systems. Figure 5 shows the energy dissipated as a function of both the applied electric current and the ethanol content of the solutions. Regardless of the membrane system, a similar behavior is observed such that the dissipated energy increases with both the electric current and the ethanol content (cell resistance).

For all the systems, the values estimated for the energy dissipation were of the order of 10^−3^ Js^−1^. An electro-osmotic experiment takes approximately one hour; it involves an energy dissipation of the order of J, and an entropy production around 10^−3^ JK^−1^, which increases with the ethanol percentage in the solution. Taking into account that the volume of solution in the chambers was approximately 4 × 10^−4^ m^3^, this energy is too low to originate an appreciable local heating of the solution, and thus, we can consider an isothermal system despite the Joule effect contribution.

### 4.3. Electro-Osmotic Permeability

**Figure 6:**
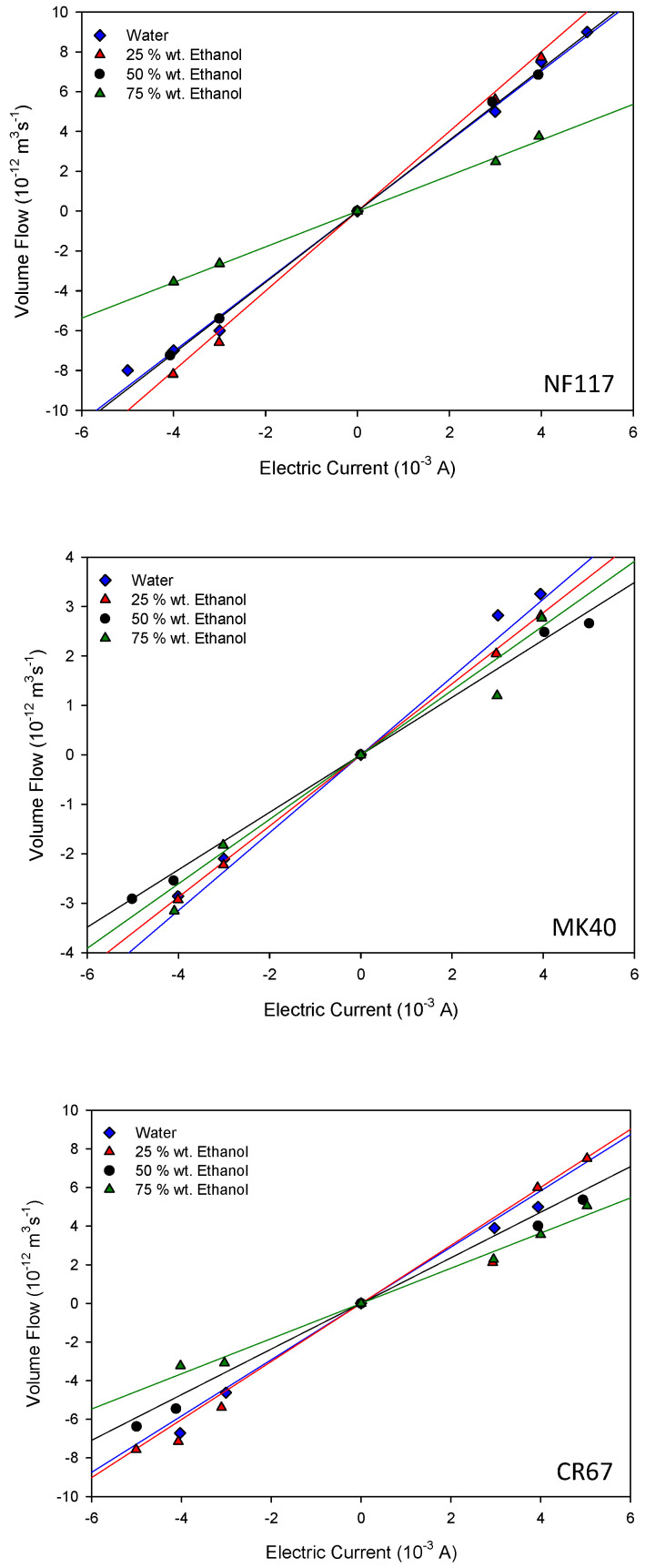
Electro-osmotic flows as a function of the applied electric current for the different membrane systems. The name of the corresponding membrane is indicated at the bottom right of the figure.

Following the method described in Section 3, the volume changes in the cathodic and anodic compartments were measured at different electric currents for all the membrane systems. Figure 6 shows the measured electro-osmotic flows as a function of the applied electric current for the different membrane systems. A similar behavior was observed in all the studied systems. After applying the electric current, a linear dependence was found between the volume change and time when the stationary state was reached. Thus, the volume flow could be estimated from the slope of the corresponding straight lines. From data in Figure 6, the values of the apparent electro-osmotic permeability *W* can be estimated using Equation (14).

Data analysis reveals that the NF117 membrane exhibits the largest electro-osmotic permeability values. For this homogeneous membrane, the variation of *W* with the ethanol content was similar to that observed for the liquid uptake, with a maximum value between 25 wt.% and 50 wt.% of ethanol in solution. Similar behavior was also observed for this membrane with methanol-water-electrolyte solutions [21]. On the other hand, both heterogeneous reinforced membranes exhibited a decrease of *W* with the ethanol percentage, with this reduction less pronounced for the MK40 membrane. In agreement with Equation (10), the corresponding pressure coefficient *β* can be estimated. The values are shown in Table 3. The obtained values in pure water are in agreement with others existing in the literature for similar membranes [5,6,7]. In these works, values of *β* of 10^−9^ VPa^−1^ were also obtained with charged membranes in aqueous electrolyte solutions. Our results were also in agreement with those obtained by Zhang et al. [25] with porous membranes in organic-aqueous solutions. They found that the larger the content of the organic solvent, the lower the streaming potential across the membrane; they attributed this behavior to the decrease of the relative dielectric constant of the solvent with increasing the ethanol content.

CR67 membrane showed larger pressure coefficients than MK40 membrane, in agreement with its larger liquid uptake. This different behavior observed for the different membranes may be due to their morphological differences since the electric properties are similar, as shown in Figure 4. Liquid uptake seems to have less influence in the pressure coefficient of highly cross-linked membranes than in non-cross linked membranes.

Wet porosity of the membrane, expressing the volume of free solution within the membrane per unit volume of wet membrane, can be obtained from its liquid uptake, *S*, by means of the expression:

(16) $$\varepsilon ={\left(1+\frac{100\rho_{l}}{S\rho_{m}}\right)}^{-1}$$

where *ρ_l_* is the density of the sorbed liquid. The values for the studied membranes are also given in Table 3. In water, membrane porosity is related to membrane density, as expected, and the higher the density, the lower the porosity. However, this relation was not observed in the presence of ethanol, indicating that the presence of ethanol affects the membrane structure. With heterogeneous membranes, a relation seems to be observed between the pressure coefficient and porosity for the same membrane, in such a way that this parameter is favored by a larger porosity. However, no relation was observed when both heterogeneous membranes were compared. No definite trend was observed with NF117 membrane. Thus, it seems that the membrane porosity would not be a decisive parameter in the pressure coefficient value, and the influence of the liquid content may be more related to its influence on the membranes’ electric properties.

**Figure 7:**
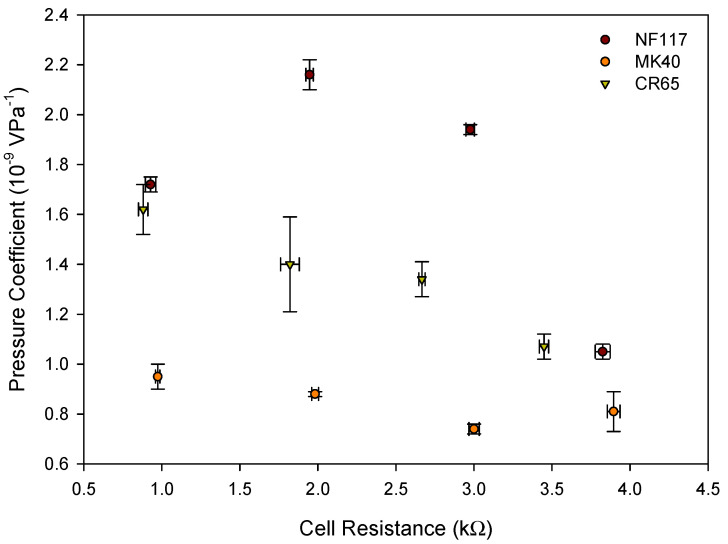
Pressure coefficient versus cell resistance for each membrane system.

Figure 7 shows the relation between cell resistance and pressure coefficient for each membrane for the different analyzed solutions. From the observed increase of the cell resistance with the ethanol percentage in the solution, an increase in energy dissipation, due to the Joule effect, is expected and thus, the pressure coefficient decreases. Both pumping mode and generation mode are electrokinetic energy conversion processes. Pumping mode involves conversion of electrical energy into mechanical energy, whereas generation mode involves a conversion of mechanical energy into electrical energy. The conversion efficiency is the same for both modes [4].

Figure 7 shows this behavior for heterogeneous MK40 and CR65 membranes, and a decrease was observed on the pressure coefficient with the increasing of the cell resistance. However, homogeneous NF117 membrane did not show a monotonous trend between both parameters, and a lower value of the pressure coefficient was observed with pure water despite the lower value of the cell resistance in the absence of ethanol. This behavior may be related to the different trend observed in the solvent content of the membrane with the percentage of ethanol, which was higher in ethanol-water mixtures than in the corresponding pure liquids for the homogeneous membrane. It is well known that the fixed charged concentration of ion-exchange membranes depends on the absorbed liquid, and that there is a strong correlation between effectiveness of fixed charge and physicochemical properties in sulfonated membranes. [26,27]. For less swollen membranes, a higher interaction between ion-pair counter-ions and fixed charged groups in the membranes is observed resulting in a larger loss selectivity. NF117 membrane showed higher solvent content when ethanol in low percentage is presented in the solution. Thus, the effective fixed charges and thus, the streaming potential, would be higher, despite the higher value of the resistance. Dense membranes would seem more appropriate for electrokinetic conversion in the presence of ethanol. However, it is necessary to take into account that hydraulic permeability is also involved in the process efficiency. This parameter is also affected by the membrane structure. Previous studies carried out with aqueous electrolyte solutions would indicate that denser membranes present lower hydraulic permeability values [28]. However, it would be necessary to study the influence of the ethanol presence.

Membrane system efficiency at converting hydrostatic potential energy to electrical power depends on its linear electrokinetic response properties [29,30]. Nowadays, the efficiency of electrokinetic energy conversion is very low and it should be increased to competitively complete the energy conversion process. The data presented show that an appropriate selection of membrane solution could increase the streaming potential value, and thus, the efficiency of the process. In this challenge a thermodynamics study of this kind of material is fundamental to estimate the corresponding electrokinetic figure of merit.

## 5. Conclusions

In this work, three commercial ion exchange membranes with different structures were compared in terms of liquid uptake and electrokinetic properties on ethanol-water media.

A different trend for the liquid uptake with the ethanol percentage was observed between homogeneous and heterogeneous membranes. Heterogeneous reinforced membranes showed a decrease in liquid uptake with an increase of the ethanol percentage on solvent. Homogeneous non-reinforced membrane showed a different trend, with an increase in the ethanol percentage for concentrations lower than approximately 50 wt.% and a decrease for higher ethanol concentrations.

The cell resistance linearly increased with the percentage of ethanol in the solution for the three membranes, probably due to a larger solution contribution to the total value. Lower values and less influence of the ethanol percentage in the solvent was observed with the more porous CR67 membrane.

The presence of alcohol in the solutions can increase, depending on the structure of the membrane and the value of the streaming potential, despite the increase in cell resistance. A higher pressure coefficient was observed with non-crosslinked homogenous membranes, mainly in the presence of low ethanol percentages, probably due to the higher solvent content observed for this kind of membrane on the measured conditions.

Nowadays, the efficiency of electrokinetic energy conversion is very low and it should be increased to competitively complete the energy conversion process. The results presented seem to indicate the influence of both membrane and medium in the pressure coefficient, and thus, in the efficiency of electrokinetic energy conversion by means of polymeric ion-exchanger membranes.

## Figures and Tables

**Table 1:** Dry thickness (*d_m_*), density (*ρ_m_*), ion exchange capacity (IEC), and average number of liquid molecules per functional group parameter (*λ*), in water and in ethanol, of cation-exchange membranes used in this study.

Membrane	*d_m_* (10^−6^ m)	*ρ_m_* (kg m^−3^)	IEC (meq g^−1^)	*λ* *	
				Water	Ethanol
NF117	181	1.98	0.94	11	2
MK40	450	1.12	1.52	15	2
CR67	685	0.833	2.1	17	2

* In 0.01 M KCl solution.

**Table 2:** Cell resistances in pure water and slopes of the cell resistance in ethanol of straight lines.

Membrane	Cell Resistance in Pure Water (kΩ)	Slope (kΩ/% wt.)
NF117	0.928 ± 0.033	0.0389 ± 0.0012
MK40	0.974 ± 0.014	0.0391 ± 0.0008
CR67	0.881 ± 0.012	0.0342 ± 0.0010

**Table 3:** Pressure coefficient (*β*) and wet porosity (*ε*) for the different membrane systems.

Membrane	Ethanol Percentage (wt.%)	*β* (10^−9^ V Pa^−1^)	*ε*
NF117	0	1.72 ± 0.03	0.27
	25	2.16 ± 0.06	0.44
	50	1.94 ± 0.02	0.47
	75	1.05 ± 0.03	0.35
MK40	0	0.95 ± 0.05	0.33
	25	0.88 ± 0.01	0.33
	50	0.74 ± 0.02	0.29
	75	0.81 ± 0.08	0.25
CR67	0	1.62 ± 0.10	0.35
	25	1.40 ± 0.19	0.35
	50	1.34 ± 0.07	0.31
	75	1.07 ± 0.05	0.27
